# The Hypothalamic–Pituitary Axis and Autoantibody Related Disorders

**DOI:** 10.3390/ijms18112322

**Published:** 2017-11-03

**Authors:** Cristina Cocco, Carla Brancia, Giulia Corda, Gian-Luca Ferri

**Affiliations:** Department of Biomedical Sciences, University of Cagliari, 09042 Monserrato, Cagliari, Italy; cbrancia@unica.it (C.B.); giuliacrd@gmail.com (G.C.); ferri@unica.it (G.-L.F.)

**Keywords:** autoimmunity, pituitary, hypothalamus, autoantibodies

## Abstract

This review summarized different studies reporting the presence of autoantibodies reacting against cells of the pituitary (APAs) and/or hypothalamus (AHAs). Both APAs and AHAs have been revealed through immunofluorescence using different kinds of substrates. Autoantibodies against gonadotropic cells were mainly found in patients affected by cryptorchidism and hypogonadotropic hypogonadism while those against prolactin cells were found in different kinds of patients, the majority without pituitary abnormalities. APAs to growth hormone (GH) cells have been associated with GH deficiency while those against the adrenocorticotropic cells have distinguished central Cushing’s disease patients at risk of incomplete cure after surgical adenoma removal. AHAs to vasopressin cells have identified patients at risk of developing diabetes insipidus. APAs have been also found together with AHAs in patients affected by idiopathic hypopituitarism, but both were also present in different kinds of patients without abnormalities of the hypothalamic–pituitary axis. Despite some data being promising, the clinical use of pituitary and hypothalamus autoantibodies is still limited by the low diagnostic sensitivity, irreproducibility of the results, and the absence of autoantigen/s able to discriminate the autoimmune reaction involving the pituitary or the hypothalamus from the other autoimmune states.

## 1. Introduction

### 1.1. The Hypothalamic–Pituitary Axis

Two endocrine organs that cooperate to control the endocrine system of the body constitute the hypothalamic–pituitary axis. In fact, the hypothalamus controls the pituitary gland (or hypophysis), which in turn, by releasing different kinds of hormones, influences the majority of the endocrine glands in the body—such as thyroid, adrenal, and gonads—as well as regulates growth, milk production, and water balance. In addition to the control of the pituitary functions, the hypothalamus also has a number of connections with the limbic lobe as well as certain areas of the extrapyramidal motor system [1,2,3]. Three lobes compose the pituitary gland: anterior, intermediate, and posterior. The thyroid-stimulating hormone (TSH), adrenocorticotropic hormone (ACTH), follicle-stimulating hormone (FSH), luteinizing hormone (LH), prolactin (PRL), and growth hormone (GH) are produced by the anterior lobe. The posterior pituitary instead releases vasopressin (ADH) and oxytocin, both produced by the paraventricular and supraoptic hypothalamic nuclei. The main function of ADH is to peripherally regulate the water homeostasis, while oxytocin is secreted in response to stimulation of the uterus during labor and nipples from the infant. ADH is also released at the median eminence level, from which reaches the anterior pituitary where it stimulates ACTH cells, together with corticotrophin-releasing hormone (CRH) to produce ACTH [4]. For its role in stimulating ACTH release, ADH seems to be a good marker of depression [5]. The pars intermedia is located between the anterior and posterior lobes of the pituitary, and produces the melanocyte stimulating hormone (MSH) in human fetal life. However, in adulthood, it is usually very small or absent. The median eminence, a crucial area for the axis located on the infundibulum, represents a connection between the hypothalamus and the hypophysis and includes the internal and the external zone. The internal zone is comprised by the axons of the hypothalamic–neurohypophyseal system containing ADH and oxytocin. The external zone instead contains numerous axons of the hypothalamic tuberoinfundibular system that releases various neurofactors. The neurofactors are secreted through the portal blood and reach the anterior pituitary, where they excite, or reduce, the hormone secretions. The growth hormone-releasing hormone (GHRH), CRH, and gonadotropin-releasing hormone (GnRH). Many axon terminals, however, contain other substances that act as modulatory factors on other axon terminals rather than secrete into the portal plexus. For instance, axon terminals containing catecholamines (dopamine, noradrenaline, and adrenaline), modulate secretion of GH through GHRH [6].

### 1.2. Diseases of the Hypothalamic–Pituitary Axis

The pituitary disorders include pituitary tumors, traumatic brain injury, hypopituitarism, hyperpituitarism, and diabetes insipidus. Pituitary tumors are typically not malignant but could affect the pituitary in its function; indeed they may generate compression causing headaches, vision difficulties, or other problems [7]. Tumors could also cause the extra production of hormones, or their decrease. Traumatic brain injury (TBI) occurs when an external power hurts the brain. It may result in pituitary dysfunction, indeed, 20–50% of the patients with TBI have pituitary dysfunctions, among which the most frequent is the GH deficiency [8]. Hypopituitarism is a condition characterized by a decrease in the normal production of one or more pituitary hormones, and, as mentioned, could be produced by pituitary tumors. With regard to the GH deficiency, it is most commonly due to pituitary adenomas and/or their treatment, even if many evidences show that also other causes are possible. Often when the cause is unknown, it is defined idiopathic. The opposite condition is the hyperpituitarism, characterized by high levels of pituitary hormones. Elevated GH blood levels, often due to tumors of the pituitary, produces acromegaly [9] while the increase in ACTH secretion stimulates the synthesis of cortisol by the adrenal glands and produces the Cushing’s disease, caused by pituitary adenomas for the 80% [10]. The diabetes insipidus is a rare condition that leads to frequent urination and excessive thirst caused by problems at the pituitary gland and/or kidneys [11]. The central diabetes insipidus is due to a lack in the ADH production, caused by neurodegeneration of the hypothalamic nuclei, with an unusually massive volume of dilute urine. About 20–50% of the cases are considered idiopathic [12].

### 1.3. Autoimmune Diseases

The autoimmune process occurs when in one individual, the cells, organs and/or tissues are attacked by their own antibodies (abs), hence named auto-abs. Consequently, all the diseases resulting from this effect are named autoimmune diseases that could be systemic or organ-specific. Systemic autoimmune diseases are characterized by the presence of auto-abs directed to non-specific tissue antigens (ags). These include, for instance, Sjögren’s syndrome, sarcoidosis, scleroderma, rheumatoid arthritis, and cryoglobulinemic vasculitis. Organ-specific autoimmune diseases instead include different organs or tissues, and could be classified as endocrinological (diabetes mellitus type 1, Hashimoto’s thyroiditis, Addison’s disease), gastrointestinal (celiac disease, Crohn’s disease, pernicious anaemia), dermatologic (pemphigus vulgaris, vitiligo), or neurological (myasthenia gravis, encephalitis). The diagnosis of autoimmune disorder is defined on the basis of accurate physical examination of the patient, associated with routine laboratory tests in order to detect specific auto-abs, by immunofluorescence (IF), or enzyme-linked immunosorbent assay (ELISA). Usually, the levels of auto-abs are biological markers of the disease development.

### 1.4. Autoimmunity and Hypothalamic–Pituitary Axis

The autoimmune inflammation of the pituitary gland is named lymphocytic hypophysitis, also defined as “autoimmune hypophysitis”. It could affect anterior and posterior lobes or both (named lymphocytic adeno-hypophysitis, infundibulo-neuro-hypophysitis, or pan-hypophysitis, respectively) [13]. Autoimmune hypophysitis is a rare disease, with a low incidence on the general population, (approximately one in nine million/year), most commonly diagnosed in women during pregnancy or postpartum or in women affected by Sheehan’s syndrome, characterized by pituitary gland necrosis, caused during or after the partum [13]. Comorbidities can also be present including thyroiditis, type 1 diabetes mellitus, and Addison’s disease. Its morphological features are suggestive of an autoimmune pathogenesis. Indeed, at histopathology, it is characterized by extensive lympho plasmacytic infiltration of T and B cells, plasma cells and occasional eosinophils, macrophages, histiocytes, and mast cells [13]. Magnetic resonance imaging (MRI) shows increase of the hypophysis with uniform enhancement involving also the hypothalamus [13]. The autoimmune process targets specific pituitary cell types, with early involvement of ACTH, FSH/LH, or TSH secreting cells causing destruction of the gland [13]. Unfortunately, definitive diagnosis can be achieved only by histology on trans-sphenoidal biopsy specimen, while non-invasive diagnosis includes MRI and endocrinological functional tests [13]. Hence, the search of pituitary auto-abs as a non-invasive diagnostic test combining low costs with simple use is ongoing.

### 1.5. Aim of the Review

The aim of this review is to summarize the relevant studies reporting the auto-abs reacting to cells of the pituitary (APAs), hypothalamus (AHAs), or both, and reveal their possible relation with alterations of the hypothalamic–pituitary axis.

## 2. Pituitary and Autoimmunity

### 2.1. APAs to Gonadotropic Cells

Auto-abs against gonadotropic cells are wholly examined in a previous review [14]. Briefly, in that review it was reported that auto-abs to gonadotropic-secreting cells were related to both cryptorchidism and hypogonadotropic hypogonadism (Table 1). APAs against gonadotropic cells were detected by IF on mammalian tissue sections and, in the majority of the cases, the auto-abs were not the pituitary hormones themselves, but unknown molecules. Despite the studies carried out so far, it is still not elucidated if the auto-abs against gonadotropic cells are the cause of cryptorchidism and hypogonadotropic hypogonadism or simply a secondary phenomenon.

### 2.2. APAs to PRL Secreting Cells

The first evidence of APAs against PRL cells was reported in the 1970s, using IF through unfixed human pituitary sections [15]. A high population was investigated (*n* = 287), including patients with different kinds of autoimmune endocrine diseases (including vitiligo, diabetes mellitus, and Addison’s disease, among others). Among the patient’s sera investigated, about 6% reacted with PRL cells (titers varied from undiluted to 1:80), however correlations with specific clinical features were not reported. Another study [16] investigated patients affected by different types of autoimmune endocrine diseases (*n* = 180, including Addison’s disease but also thyroid related alterations and central diabetes), using sections of unfixed baboon pituitary. The presence of APAs at a low titer (1:8) was revealed in 22% of the cases. Afterwards, some of APA-positive sera were re-investigated and identified to be directed exclusively to PRL containing cells [17]. As in the previous study, pituitary abnormalities were not reported in any of the APA positive patients. Hence, it was concluded that the presence of APAs against PRL cells at a low titer in patients with autoimmune diseases could be just a non-specific manifestation. Instead, puerperal alactogenesis was related to PRL-cell-auto-abs in a 39-year-old woman [18]. Indeed, auto-abs directed against PRL cells, but not against the PRL hormone itself, were revealed in parallel with undetectable PRL blood levels. The patient’s serum was incubated (diluted 1:10) on sections of unfixed human pituitary collected at autopsy. When analysis of the calcium levels and cranial MRI was applied, both were found normal. Moreover, genetic analysis showed that there were no rare sequence variants in the *PRL* genes. Hence, in this rare case, it was suggested that a pituitary autoimmune process was involvement in the PRL deficiency, confirmed by the fact that the exogenous PRL treatment produced a total resolution of the problem [18]. Patients affected exclusively by a thyroid autoimmune condition named Grave’s disease (*n* = 22) were studied by an immunocytochemical tissue assay at dilution 1:100, using rat and swine pituitaries. APAs were found directed exclusively to PRL cells (in three patients), or against both PRL and GH cells (in two further patients), but they were also present in 9.2% of the healthy controls (*n* = 97) [19]. However, the pathological significance of these auto-abs was not elucidated. Interestingly, PRL cell auto-abs were also found in patients affected by neurological diseases (Alzheimer’s and Down’s syndrome) [20]. Indeed, patient’s sera were used undiluted through unfixed human pituitary and APAs were found in 26 out of 27 Alzheimer’s disease patients with dementia as well as in 10 out of 11 patients with Down’s syndrome with dementia [20]. However, when the same study was repeated by another group [21], only 2 out of the 23 sera from patients affected by Alzheimer or Down’s syndrome, revealed APAs against PRL cells. In the same study, pituitaries from monkey, baboon, and human were compared, and the monkey sections resulted in a major number of labelled cells. The role of these auto-abs in the etiopathogenesis of Alzheimer’s disease and Down’s syndrome was not demonstrated. In conclusion, PRL cell auto-abs have been rarely associated with pituitary abnormalities, hence their significance remains unclear. Studies reporting the presence of PRL auto-abs have been summarized in Table 1.

### 2.3. APAs to GH Secreting Cells

The presence of the auto-abs directed to GH cells have been investigated to examine their possible role in the development of partial and/or idiopathic GH deficiency (Table 1). The first reported case was a girl with Turner’s syndrome affected by partial GH deficiency, while the other pituitary hormones were normally secreted [22]. The serum was incubated on sections of unfixed human pituitary and APAs were revealed (at low titer, 1:8) exclusively directed against GH cells suggesting these abs as serological markers for GH-cell destruction. Another study [16], aimed to better examine by IF on unfixed baboon sections, the presence of APAs in an adult population either affected by GH deficiency (*n* = 26) or different types of autoimmune endocrine diseases (including patients affected by Addison’s disease, thyroid autoimmune conditions and central diabetes, total *n* = 180). The adult patients with GH deficiency were subdivided into two groups: one with isolated and apparently idiopathic GH deficiency, treated with recombinant GH during childhood (*n* = 12), while the other group was constituted by patients with GH deficiency secondary to surgery for pituitary and parasellar tumors (*n* = 14). APA were found at high titers (1:32–64) in patients with idiopathic GH deficiency (33%), but not in those affected by GH deficiency secondary to surgery. Moreover, APAs were also found at a low (1:28) or high (1:32–64) titer in about 22% of the patients with organ-specific autoimmune diseases. Regarding the pituitary function, interestingly, all APA-positive patients at high titers had a severe GH deficiency, while the 20 APA negative patients with autoimmune endocrine diseases and the patients with APAs at low titers; all of them had a normal pituitary function. Moreover, an opposite relationship between the APA titers and the GH peak (evaluated by the insulin tolerance test) was observed in patients with autoimmune endocrine diseases. All these data suggest that high titer APAs could be associated with GH deficiency in adults with either autoimmune endocrine diseases or apparently idiopathic GH deficiency. Later on [17], in order to indicate which kind of pituitary cells were involved in this autoimmune reaction, the same adult patients without pituitary impairment and affected by autoimmune endocrine diseases containing APAs at a low titer (<1:8) were re-investigated, together with patients containing APAs at a high titer (>1:8) and affected by apparently idiopathic GH deficiency. This group was subdivided into patients diagnosed during childhood (*n* = 4, group 1) or adulthood (also associated with autoimmune endocrine diseases; *n* = 5, group 2). APAs directed against the GH cells were exclusively found in those patients affected by isolated GH deficiency diagnosed during childhood, while, in those diagnosed in the adulthood, they were revealed together with another kinds of APAs and rarely found in those patients affected by autoimmune endocrine diseases. The reason of the autoimmunity against multiple kinds of pituitary cells in those patients diagnosed in the adulthood was not understood. Auto-abs against GH cells (1:2000–10,000 dilutions), were also found using fixed guinea pig pituitary sections in three out of six APECED (autoimmune polyendocrinopathy-candidiasis–ectodermal dystrophy/dysplasia) patients with GH deficiency (age range: 6–18 years), while the controls (*n* = 10) were negative [23]. Interestingly, after pre-adsorbed experiments, in one case, the staining was abolished with the Aromatic l-amino acid decarboxylase enzyme, that is one of the peculiar circulating auto-ags in APECED [23]. Auto-abs against GH cells were also revealed in patients with neurological disorders. Indeed, 22 patients affected by multiple sclerosis were studied through IF on fixed rat and hog pituitary sections (dilutions: 1:400–3200), and 50% of them demonstrated a reaction at a low (<1:600) or high (>1:600) titer [24]. However, in these patients, association between APAs and specific clinical manifestations has not been found. In conclusion, it seems that GH deficiency is highly associated with GH auto-abs.

### 2.4. APAs to ACTH Cells

Circulating auto-abs to unfixed human fetal pituitary cells were detected in 13 out of 51 patients (vs. 51 age-matched control subjects) who underwent trans-sphenoidal microsurgery for Cushing’s disease [25]. In all the 13 patients, auto-abs were revealed before surgery, and 10 of them showed clinical and/or biochemical signs of incomplete cure of Cushing’s disease after surgery. However, also 3 out of those 27 patients with a favourable outcome were APA positive. The APA titers were comprised between 1:2 and 1:8. In all the positive patients, the auto-abs were directed to ACTH-producing cells and, in eight of them, also directed to other kinds of cells (GH and LH). Hence, the presence of ACTH-cell-auto-abs in patients with Cushing’s disease was suggested to be associated with an unsuccessful outcome after surgery [25]. In one adolescent girl with Turner syndrome, APAs were suggested to be associated with her secondary adrenal insufficiency [26]. This 15-year-old girl had hypothyroidism, and ovarian failure with hypocortisolism and secondary adrenal insufficiency, hypotrophic pituitary gland and loss of the posterior pituitary bright spot. The patient’s serum contained auto-abs (dilution 1:10) that recognized 36% of the ACTH cells, as well as the majority of the gonadotropic-secreting cells but not the pituitary hormones themselves. Studies reporting the presence of ACTH auto-abs have been summarized in Table 1.

### 2.5. APAs: Other Studies

Several studies involving large populations of subjects, report that circulating APAs are present in patients affected by diabetes mellitus type 1 (Table 1). Undiluted and diluted (1:4–200) sera incubated on cryostat sections of unfixed human and monkey, as well as fixed rat and bovine pituitaries were used through [27,28,29,30]. Sometimes, APAs were directed to multiple types of pituitary hormone cells [27] or have been reported without specific references regarding the cell type(s) involved [28,29,30]. The APA incidence was highly different between the studies and the results were often discordant. In one study [30], 11 diabetes mellitus type 1 sera, previously analyzed for APAs using monkey sections, were subsequently tested on bovine sections while vice versa 22 diabetes mellitus type 1 sera found APA positive on bovine sections were re-tested on monkey sections. However, none of the APA-positive sera on bovine substrate were confirmed positive on monkey substrate and vice versa. Hence, taken together, all these data show that not only the incidence of APAs in diabetes mellitus type 1 is variously reported, but also the results are not always reproducible. APAs were also investigated using IF on cryostat sections of rat pituitaries in a small population of patients affected by ACTH deficiency (*n* = 21, Table 1), and found in about 47% of them [28]. Another big study (Table 1) involving a large number of subjects has examined, using IF and unfixed young baboon as substrate, the presence of APAs in patients with autoimmune thyroid diseases (*n* = 961, AITD) and no AITD (*n* = 329) in comparison with control subjects (*n* = 135) [31]. APAs were more present in patients with AITD (11.4%) compared to those with non-AITD (0.9%) but they were not revealed in the control subjects. The majority of APA positive patients (*n* = 102) were submitted to dynamic testing for functional pituitary assessment, and 35.2% had mild or severe GH deficiency with pituitary abnormalities revealed with MRI. Hence, in conclusion, this big study shows that APAs, in AITD patients, are highly associated with GH deficiency and pituitary abnormalities. APAs were also revealed by IF [32], in 13 out of the 29 sera from patients with TBI (44.8%), but in none of the control sera (Table 1). Pituitary dysfunction development ratio was significantly higher in APA-positive patients (46.2%) when compared with APA-negative ones (12.5%) and there was a significant positive correlation (*r* = 0.74, *p* = 0.004) between the APA titer ratio and the low GH response. Hence, it was suggested that autoimmunity might contribute to the development of TBI-induced hypopituitarism [32].

### 2.6. Pituitary Auto-Ags

The search of pituitary auto-ags has been very extensive, carried out through different techniques. Indeed, through the immunoblotting on human pituitary, different bands have been revealed in sera from Graves’ disease patients, including those of 49 and 40-kDa [33]. APAs reactive to a 49-kDa pituitary cytosolic protein were found in 70% of biopsy-proven lymphocytic hypophysitis, 55% of suspected hypophysitis, but also in other diseases including Addison’s disease (42%), and pituitary tumors (20%), as well as in 9.8% of normal subjects [34]. Reactivity to a 40-kDa cytosolic protein was also found in 50% of patients with biopsy-proven disease [34]. The form of 49-kDa was then identified with α-enolase [35], a ubiquitous enzyme present in most tissues and organs, not only in the pituitary. Moreover, the full-length α-enolase was revealed also as auto-ag in other kinds of patients, including those with pituitary adenomas (6/13, 46%), other autoimmune diseases (6/30, 20%), and even healthy controls (2/46, 4%) [35]. In another interesting study, 28 sera from autoimmune hypophysitis patients (14 histologically proven and 14 suspected) were compared to 98 sera from controls (included 14 patients with pituitary adenomas, 48 with autoimmune thyroiditis and 36 healthy subjects) using a combined approach of immunoblotting and mass spectrometry, as well as IF on monkey pituitary sections [36]. A region of 25–27 kDa was highly associated to the hypophysitis cases and encompassed two novel possible auto-ags: the C14orf166 and chorionic somatomammotrophin proteins [36]. Unfortunately, despite the innovative method, this test did not allow discrimination of the auto-immune hypophysitis from other autoimmune states. Through the use of a pituitary cDNA expression library, a large cohort of patients (86 with hypophysitis and 90 controls) was investigated and the pituitary gland-specific factor 1a (TPIT), was identified as auto-ag in 10.5% of patients with hypophysitis [37], while PGSF-1a and -2 and neuron-specific enolase, were also found, but at a frequency that did not differ from that of the healthy controls [37]. However, since TPIT auto-abs were also detected in patients with other autoimmune endocrine diseases, they are not specific for lymphocytic hypophysitis. Among the APECED patients investigated (*n* = 86), a single protein namely “Tudor Domain Containing Protein 6” (TDRD6) was identified as auto-ag in 42 cases [23]. However, TDRD6 is largely expressed not only in the pituitary but also in the testis, adrenal gland, and pancreas and its function remains unknown. Taken all together, these data show that, unfortunately there is no a valuable test based on a specific auto-ag that can discriminate the autoimmune hypophysitis from other autoimmune states.

## 3. Hypothalamus and Autoimmunity

Using IF on unfixed tissue samples of human (from both fetal and adults) and baboon hypothalamus, AHAs to ADH cells (titer: 1:1–32) were reported in adult patients affected by diabetes insipidus, in both idiopathic (11 out of 30) and symptomatic (2 out of 32) cases, but in none of the controls (*n* = 139) [38]. Human fetal was suggested to be the best substrate. This study demonstrates that autoimmunity can also be extended to the hypothalamus and it reveals the importance of AHAs against ADH cells as markers of an autoimmune type of diabetes insipidus [38]. Analogous results were obtained when children with idiopathic central diabetes insipidus (*n* = 12) were investigated in parallel with patients affected by Langerhans cell histiocytosis (*n* = 6) and germinoma (*n* = 2) [39]. Indeed, 75% of them had AHAs to ADH cells, five of them showed a constant positivity throughout the follow-up while one became AHA positive afterwards. The analyses to determine the pituitary function were applied and pituitary abnormalities were shown in the majority of the AHA positive children. However, AHAs to ADH cells were also revealed in four patients with Langerhans cell histiocytosis and two with germinoma, denoting the possibility that they could not be specific for central diabetes insipidus. When auto-abs against ADH cells were investigated in autoimmune endocrine disease patients without central diabetes insipidus (*n* = 410 in total, *n* = 260 with thyroid autoimmune disease, and 150 with insulin dependent diabetes mellitus; vs. 100 normal subjects), none of the controls, but 5 out of 410 patients (1.2%) were positive for ADH cells (sera were used either undiluted or diluted 1:40) [40]. All positive—as well as nine negative—patients were analysed for the posterior pituitary function, and two out of the five with ADH auto-abs had partial central diabetes insipidus [40]. These data could indicate the existence of a subclinical phase preceding the clinical diabetes insipidus characterized by the presence of the AHAs against ADH cells. An autoimmune reactivity has also been reported as a consequence of the therapy involving the extended use of animal proteins [41]. Indeed, a serum from a patient who had been treated with pitressin for an extended period, was evaluated for the presence of auto-abs by IF (dilution 1:100) or IP (1:400) on rat tissues. AHAs were found directed against neurophysin-containing cells of the rat paraventricular, supraoptic, and suprachiasmatic hypothalamic nuclei [41]. Autoimmunity at the hypothalamic level has been also revealed in patients affected by psychiatric diseases [42]. Circulating auto-abs (dilution 1:10), assessed by IF, enzyme linked immunosorbent assay and chemiluminescence immunoassaies, were found reacting to various rat brain areas including the hypothalamus (through a BioSystem kit) in sera from patients with schizophrenia (4 out of 30), mood disorders (11 out of 20), and in some healthy controls (2 out of 39). These data underline the concept that an autoimmune process involving the hypothalamus could be related also to affective disorders, even if the physiopathology of these auto-abs remains to be elucidated [42]. All data regarding AHAs have been summarized in Table 2.

## 4. Hypothalamus and Pituitary Autoimmunity

The presence of both APAs and AHAs have been examined in patients affected by idiopathic hypopituitarism, traumatic brain injury with hypopituitarism, celiac disease, and Sheehan’s syndrome with pituitary dysfunctions (Table 3). Patients’ sera were investigated through IF on unfixed baboon pituitary and hypothalamus. Among the 66 patients affected by idiopathic hypopituitarism, APAs were present at high titer (1:32–128) in 13 patients (19.6%) with pituitary dysfunctions including hypogonadotropic hypogonadism as well as ACTH and GH deficiencies, largely targeting the corresponding hormone cells, while exclusively AHAs were found at high titer in five patients with ACTH deficiency, mostly targeting CRH–secreting cells [43]. When sera from 61 male boxers were analysed (44 competing and 17 retired), AHAs were detected in 13 (21.3%), and APAs in 14 (22.9%) of them; but in none of the 60 controls. When pituitary hormonal parameters were investigated, AHA-positive boxers (46.2%) had higher dysfunctions than AHA-negative (10.4%), but there was no significant association between APA positivity and hypopituitarism [44]. Celiac children (*n* = 31, 6 with and 25 without growth deficiency) were analysed in parallel with 58 healthy controls. Those with reduced growth had APAs (4 out of the 6) at high titers (>1:8) and 2 of them were positive also for AHAs. Moreover, APAs were positive at low titers (<1:8) in 12% of those without growth deficiency but also in 2 out of the 58 controls. Hypothalamic–pituitary MRI was normal in all patients [45]. When women with Sheehan’s syndrome (*n* = 20, disease interval: 3–40 years) were investigated, even many years after the disease onset, 35% of them revealed APAs (titer: 1:16 to 1:32) while 40% showed the presence of AHAs (titers: 1:32 to 1:128) not directed to ADH-secreting cells [46]. Despite these AHAs were not characterized, this study was useful to suggest that autoimmunity at both hypothalamus and pituitary levels may be involved in the late pituitary dysfunction found in Sheehan’s syndrome patients. Using fixed hypothalamus and pituitary from bovine, APECED patients were analysed (*n* = 14) together with 23 healthy control subjects and 7 patients with idiopathic GH deficiency [47]. Half of the analysed APECED sera revealed APAs (titer ranging between 1:50 and 1:2000) directed against heterogeneous pituitary cells. Sera from healthy controls, and from patients with idiopathic GH deficiency resulted either negative or positive at a very low dilution (1:8). With the aim of comparing bovine and monkey tissues, four out of the seven APECED positive sera on bovine pituitary were also re-tested on monkey pituitary slides, resulting all in positive staining but with lower titers (titers 1:8–1:100). Interestingly, 4 out of 14 APECED patients had both APAs and AHAs directed against GHRH- (titer: 1:200–600) and/or TH- (titer: >1:4000) neurons of the bovine median eminence. Interestingly, among the patients with GH deficiency (*n* = 5), 4 had APAs and/or AHAs, including those labelling GH and GHRH (one directed against the hormone itself) [47]. In another interesting study, APAs and AHAs have been found in 42 out of 57 patients affected by eating disorders such as anorexia (AN) and bulimia (BN) nervosa [48]. Auto-abs were revealed by IF (dilutions: 1:200–5000) using pituitary and brain from rats treated with colchicine and perfused. In AN and/or BN patients, APA labelled melanotropes in the intermedia and/or ACTH cells in the anterior lobe, three of them (AN patients) labelled LHRH terminals in the median eminence, and were directed against the LHRH itself, in two cases. About 16% of the control subjects showed staining patterns similar to the patient sera. This study shows that an autoimmune process at the hypothalamic–pituitary axis level could occur also in eating diseases as AN and BN, despite the pathophysiological role of the above auto-abs has to be clarified [48]. Interestingly, some authors believe that ACTH auto-abs are plausible as cause of the HPA deregulation, leading to clinical symptoms present in chronic fatigue syndrome, AN and major depression [49]. In another study using paraffin embedded samples of pituitaries and brains from rats and hogs, patients affected by multiple sclerosis (*n* = 33) were investigated and 11 of them revealed APAs (titer: 1:400–3200) and AHAs (titer: 1:400–5000) directed to cells containing peptides of the somatotropin family and/or ADH/oxytocin, respectively [50]. However, using various techniques (absorption experiments, immunocytochemical model assays, and radioimmunoassays), none of the mentioned substances were revealed as auto-ags [51]. The authors hypothesized that these auto-abs might be involved in the disease demyelinization or immunoregulation, or alternatively, they may simply be a secondary phenomenon of the disease.

## 5. Conclusions

### 5.1. Technical Considerations

The main approach used to reveal auto-abs includes IF that remains a widely used technique to reveal the precise location of the auto-ab reactivity within specific cell type/s of the hypothalamic–pituitary axis, combining low costs with simple use. However, through IF, the interpretation of results is often difficult due to the presence of positive reactivity often revealed also in sera from control subjects. In our opinion, a good approach is to study a high number of sera from control subjects (about 100) from which to get a threshold signal using dedicated software [52]. The human tissue has been indicated as the most compatible [25]. However, due to its low availability and inadequate quality, many researchers have opted for other mammal tissues, including guinea pig [23], rat, bovine [47,48,52,53], baboon [16,17], and monkey [29,30]. Unfixed or fixed tissues have been used throughout, while in our opinion both approaches should be used, at least in pilot experiments, in order to have the highest possibility to reveal unknown ags. Indeed, in some cases, the unknown ag could be masked by the fixation, but in other cases, it could be much better revealed with a good fixation. Another important point to consider is the auto-ab titer, indeed, using human, baboon or monkey as substrates, the titers are always lower than those obtained using rats or other mammalians. In our opinion, this difference could be due to the low preservation of the tissue, especially in the case of human tissues.

### 5.2. Can APAs and/or AHAs Be Used in the Routine Clinical Practice?

Taken together, all data indicate the existence of limitations that preclude the use of AHAs and APAs as markers of autoimmune diseases of the hypothalamic–pituitary axis. First of all, they have been detected more often in patients with normal function and/or intact anatomo-morphology of the hypothalamic–pituitary axis than in patients with abnormalities of the pituitary gland or hypothalamus. Secondly, the auto-ab assay is often un-reproducible, with discordant results. In our opinion, in order to be extremely confident about the real presence of the auto-ab labelling, different substrates should be used in parallel; as well as results obtained with IF should be confirmed with other techniques (western blot, ELISA). Last but not least, one important limitation remains the absence of specific auto-ag/s. The identification of a specific auto-ag could help not only in understanding the pathophiological role of the auto-abs against hypothalamic–pituitary axis, but also in the search of the appropriate substrate, in view of the possible presence of specie-specific differences in the ag sequence. The limits that we mentioned here seem to be strictly related to the hypothalamic–pituitary axis [54], whereas anti gastric parietal cell abs are associated with a type of chronic gastritis [55], and adrenal auto-abs are useful markers in predicting the onset of idiopathic Addison’s disease [56]. For all these reasons, diagnosis of autoimmunity at the level of pituitary and/or hypothalamus with a specific serological test is still not available and the present data are too insufficient to suggest specific analyses.

## Figures and Tables

**Table 1:** Incidence of APAs in autoimmune and no-autoimmune diseases.

Cell Types	Disease	No.	Incidence (%)	Dil.	Ref.
**LH/FSH**	Cryptorchidism	46	56.5	ud–1:2	[14]
	Idiopathic hypopituitarism ^1^	44	96.4	1:8–128	[14]
**PRL**	Autoimmune diseases ^2^	287	6.6	ud–80	[15]
	Puerperal alactogenesis	1	-	1:10	[18]
	Graves’ disease	22	36.36 *	1:100	[19]
	Alzheimer’s	27	96.30	ud	[20]
	Down’s syndrome ^3^	11	90.90	ud	[20]
	Down’s syndrome ^3^ and Alzheimer’s	23 **	8.6	ud	[21]
**GH**	Turner’s syndrome	1	-	1:8	[22]
	Idiopathic GHD (adults)	12	33.3	1:2–64	[17]
	Graves’ disease	22	36.3 *	1:100	[19]
	APECED with GHD	6	50	1:2 k–10 k	[23]
	Multiple sclerosis	22	50	1:400–3200	[24]
**ACTH**	Cushing’s ^4^	51	25.5	1:2–8	[25]
	Turner’s syndrome ^4^	1	-	1:10	[26]
**Multiple**	Diabetes mellitus type 1	226	18.6	ud-1:4	[27]
**N.P**	Diabetes mellitus type 1	81	29.6	ud	[28]
	Diabetes mellitus type 1	111	3.6	1:10–90	[29]
	Diabetes mellitus type 1	100	7	1:200	[30]
	ACTH deficiency	21	47.6	ud	[28]
	AITD	961	11.4	1:10–90	[31]
	Non-AITD	329	0.9	1:10–90	[31]
	Traumatic brain injury	29	44.8	1:8–256	[32]

No.: patient’s number; Dil.: dilution; Ref: reference; LH/FSH, PRL, GH, ACTH: luteinizing /follicle-stimulating, prolactin, growth and adrenocorticotropic hormones, respectively; APECED: autoimmune polyendocrinopathy-candidiasis–ectodermal dystrophy; GHD: GH deficiency; AITD: autoimmune thyroid diseases; N.P: not provided, uncharacterized cells; ud: undiluted; k = 1000. ^1^ including: 21 patients with normal sense of smell, 10 with Kallmann’s syndrome, and 13 with other pituitary hormone deficiencies; ^2^ with one or more autoimmune diseases; ^3^ affected also by dementia; ^4^ the patient showed also other auto-antibodies; * incidence including the 22% of patients with auto-antibodies reacting also to PRL cells; ** number includes 3 patients with Down’s syndrome and 20 with Alzheimer’s disease.

**Table 2:** Incidence of AHAs in autoimmune and no-autoimmune diseases.

Disease	No.	Incidence (%)	Dilution	Ref.
Diabetes Insipidus (adult)	62	43	1:1–32	[38]
Diabetes Insipidus (children)	12	75	1:40	[39]
Langerhans cell histiocytosis	6	66.6	1:40	[39]
Germinoma	2	100	1:40	[39]
Autoimmune diseases *	41	1.2	ud/1:40	[40]
Pitressin treatment	1	-	1:100	[41]
Schizophrenia	30	13.3	1:10	[42]
Mood disorders	20	52	1:10	[42]

No. = number of patients; Ref.: reference; * without central diabetes insipidus; ud: undiluted.

**Table 3:** Incidence of APAs and AHAs in autoimmune and no-autoimmune diseases.

Disease	No.	APAs (%)	AHAs (%)	Dilution	Ref.
Idiopathic hypopituitarism	66	19.6	10	1:32–128	[43]
Traumatic brain injury	61	22.9	21.3	1:8–256	[44]
Celiac children	31	12.9	6.45	1:2–64	[45]
Sheehan’s syndrome	20	35	40	1:32–128	[46]
APECED	14	50	50	1:50–4 k	[47]
(with GHD)	5	40	60	1:50–4 k	[47]
Eating disorders	57	74	20	1:200–5 k	[48]

No. = number of patients; APAs: anti-pituitary-auto-antibodies and AHAs: anti-hypothalamic-auto-antibodies; Ref.: reference; APECED: autoimmune polyendocrinopathy-candidiasis–ectodermal dystrophy, GHD: growth deficiency; k = 1000.
